# Perceived Functions of Playfulness in Adult English as a Foreign Language Learners: An Exploratory Study

**DOI:** 10.3389/fpsyg.2021.823123

**Published:** 2022-01-24

**Authors:** Elyas Barabadi, Majid Elahi Shirvan, Mojdeh Shahnama, René T. Proyer

**Affiliations:** ^1^Department of Foreign Languages, University of Bojnord, Bojnord, Iran; ^2^Department of Psychology, Martin Luther University of Halle-Wittenberg, Halle, Germany

**Keywords:** playfulness, fun and laughter, creativity, intellectual playfulness, other-directed playfulness, EFL learning

## Abstract

Influenced by the flowering of positive psychology in the field of foreign language acquisition research in recent years, the present study aimed to explore the perceived functions of playfulness, as a personality construct, among English as a foreign language (EFL) learners. To this aim, an initial sample of 38 EFL learners were selected randomly from the private language institutes of Mashhad, the second largest city in Iran. They were interviewed about any perceived functions of playfulness in the EFL learning context. A qualitative content analysis of the collected data led to the extraction of four categories: Fun and laughter, creativity, mastery orientation, and cultivating relationships. A further in-depth analysis of the categories and comparison with the functions of adult playfulness in psychology (primarily Proyer’s 2014 and 2017 works of research) revealed that these four categories can be subsumed under two of the four structural components of adult playfulness; namely, other-directed playfulness and intellectual playfulness. The ability of EFL learners to turn language learning situations, tasks, and environment into enjoyable ones via their playfulness can have implications for the quality of their interpersonal interactions in class and eventually their language proficiency. The findings of this study can pave the way for the translation of the adult playfulness construct from developmental and personality psychology and family relations into the second language acquisition (SLA) domain and its conceptualization in future research in this domain.

## Introduction

In recent years, the field of second language acquisition (SLA) has shown growing interest in positive psychology; thereby, highlighting individuals’ strengths and attributes and focusing on the elicitation of positive emotions like foreign language enjoyment (MacIntyre and Mercer, 2014; Dewaele et al., 2019). With a focus on these aspects, positive psychology can help individuals construct self-determined motivation, positive emotions, and greater engagement (Seligman et al., 2006), all of which are at the core of SLA studies. Maclntyre (2016) even argued that the advent of positive psychology in the SLA domain is overdue if we consider the focus of the field on successful communication among learners, and the personal development of language learners. The growing positive psychology research in SLA has recently explored both, negative emotions such as boredom (e.g., Kruk and Zawodniak, 2020; Pawlak et al., 2020a,b) and positive emotions like enjoyment (e.g., Dewaele et al., 2019; Li et al., 2020; ElahiShirvan and Taherian, 2021); however, the contributing role of playfulness as a personality construct that potentially can enable language learners to (re-)frame everyday experiences or boring classroom situations to enjoyable, stimulating, and/or interesting ones has not yet been addressed in this line of research. For narrowing this gap in the literature, this initial study explored playfulness in the SLA domain; more specifically, among language learners.

Playfulness is an individual differences variable that enables individuals “to frame or reframe everyday situations in a way that they experience them as entertaining, and/or intellectually stimulating, and/or personally appealing” (Proyer, 2017, p. 93). Early studies found that a higher level of playfulness among adults is associated with a better academic performance (Proyer, 2011), physical and mental health and satisfaction with life (Proyer, 2013), higher creativity (Proyer et al., 2019), stress coping (Barnett, 2011), positive emotions, better emotion regulation (Fredrickson, 2001) and positive behavior and perception at work (Yu et al., 2007) to name just a few findings. Importantly, it has been shown that people in different contexts and domains (i.e., leisure, work, work colleagues, partnership, and friends) can experience various functions of playfulness (Proyer, 2014; *N* = 324 German-speaking adults aged 18–62). Using a qualitative approach this study has shown that lay people’s perceptions about playfulness in their daily lives could largely be grouped into seven categories (the percentage gives the frequency of nominations): (1) *humor and laughter* (34.9%; e.g., to have fun, to fool around, to make others (and myself) laugh); (2) *creativity* (14.7%; e.g., to spend my time in a more interesting way; to create new things; to pursue my hobbies; to find solutions and solve problems); (3) *manage und cultivate relationships* (13.5%; e.g., to cultivate relationships; to communicate with others; to socialize; to show affection; (4) *well-being* (13.0%); e.g., to be happy; to increase my well-being; to make me feel good; to experience pleasure); (5) *coping with situations* (11.6%; to loosen up a situation; (6)*mastery orientation* (6.7%; e.g., to motivate myself; to motivate others; to be informed; to be active; and (7) *coping-self* (5.5%; e.g., to unwind; to relax; to recharge; to cope with stress; to escape daily routine). While there were differences among the domains, these seven broader categories show that playfulness seems to have a more differentiated role in the life of adults than only to enable entertainment. Although the perceived functions of playfulness have been investigated among lay persons (Proyer, 2014), to the best of our knowledge, no research has explored the perceived functions of playfulness among English as a foreign language (EFL) learners.

With this caveat in mind and in line with the growth of positive psychology in SLA, using a qualitative research design, in the current study we aimed to uncover the perceived functions of playfulness among Iranian language learners and, thereby, extending earlier findings from a German-speaking country (Proyer, 2014) to (a) a different cultural context and (b) a more selected group than the general population. To play and be playful has the potential to elicit positive emotions. Given that emotions abound in the context of language teaching and learning and given that these emotions affect the process of language learning and language achievement (Dewaele and Li, 2020) the interplay with playfulness warrants further research. A deeper understanding of the nature of playfulness and its perceived functions in the context of language learning can potentially help in the development of positive interventions aimed at facilitating the language learning process by knowing how to cultivate positive emotions and lower negative emotions (Li et al., 2021). Thus, the present study aimed to answer the following research question: *What are the potential functions of playfulness as perceived by EFL learners*?

## Review of the Literature

Barnett (2007, 2012) suggests that playful individuals are capable of (re-)framing almost any everyday situation into something entertaining, stimulating, and enjoyable (e.g., using cognitive restructuring or using imagination). One might argue that playfulness as an individual differences variables more a quality of mind and mindset that predisposes people to reframe uninteresting contexts into amusing ones. There is research evidence that individuals high in playfulness are well aware of leisure opportunities and are less likely to feel bored in doing such leisure activities compared to their less playful peers (Barnett, 2011).

There is robust evidence for a positive correlation between playfulness and many positive outcome variables such as relationship satisfaction, well-being, creativity, levels of self-estimates and self-confidence (e.g., Yu et al., 2007; Barnett, 2011, 2012; Magnuson and Barnett, 2013; Proyer, 2013; Yue et al., 2016; Proyer and Brauer, 2018; Proyer et al., 2020). Proyer (2012) investigated the link between playfulness and various measures of personality and ability (e.g., ingenuity) in a sequence of three studies: Adult playfulness is positively related to gelotophilia (the joy in being laughed at), extraversion, and higher endorsement of culture (in terms of the Five Factor Model of personality), it relates to intrinsic goals rather than extrinsic goals, and is positively correlated with both self-perception of ingenuity and psychometrically measures ingenuity. Positive associations with the engaged life (in terms of the Orientations to Happiness-model) may also point to a greater inclination to flow-experiences and the experience of positive emotions.

Proyer (2017) put forth a structural model of playfulness (OLIW-model) comprised of four aspects: other-directed (e.g., preferring to interact with others playfully, for example by reducing tensions using humor), lighthearted (e.g., taking life easy and thinking of it more as a playground rather than a battlefield; liking to improvise), intellectual (e.g., showing a tendency to play with ideas and to get involved in complex and challenging tasks: preferring complexity over simplicity), and whimsical playfulness (e.g., noticing and enjoying unusual people, things and activities. Also, Proyer et al. (2019) pointed out that playfulness relates to creativity, innovation, and divergent thinking (see also Bateson and Martin, 2013).

Studying playfulness in the context of language learning may also interest from an intervention perspective. Using adaptations of activities developed in online positive psychology intervention studies, Proyer et al. (2020) employed a set of interventions with the aim of stimulating playfulness and examining its effect on self-reported happiness and depressive symptoms. The results of both immediate and delayed post-tests indicated that all interventions could enhance not only different aspects of playfulness but also well-being by lowering depressive symptoms in the short term. Hence, such activities could potentially be used for helping language learners to manage experienced emotions and further stimulate the learning experience.

As reviewed above, the existing literature has strongly supported the positive role of playfulness not only in daily life but also at work and academic contexts. These potential benefits of playfulness arise from various functions it serves in different domains. Several studies (Proyer, 2011; Magnuson and Barnett, 2013; Proyer et al., 2020) suggest that playfulness affects different settings including academic contexts, though in a unique way. Like other psychological constructs in educational contexts such as motivation and anxiety, it seems that individuals’ playfulness is to some extent context and situation-specific, and it is not entirely an internal variable. Therefore, understating what adults perceive as functions of playfulness requires special attention in language learning context too. According to Proyer (2014), perceived functions refer to individuals’ implicit assumptions and beliefs they hold about the potential functions of playfulness. In other words, perceived functions are in fact everyday observations individuals have about playfulness, or the possible ways playfulness might make a valuable contribution to their life, including academic life. The present study allows for a comparison of the findings from Proyer (2014) using a sample of adults from the general population to a sample of EFL learners from a different cultural context.

## Materials and Methods

### Participants and Setting

The sample comprised 38 Iranian male and female EFL learners from the five most well-known national private language institutes in Mashhad, the second metropolis of Iran. We followed the principle of maximum variation (Creswell, 1998) to select the participants of the study and gave full consideration to their differences in gender, age, and years of learning experience. The participants’ age ranged between 18 and 25 years (*M* = 22, *SD* = 1.38). Among them, 20 were female (52.63%) and the rest were male (47.37%). They all shared the same nationality (Iranian), the same L1 (Persian), and all learned English as a second language. The data were collected in summer 2021.

### Instrumentation

Given the exploratory nature of this study, to collect the required data, the participants were interviewed to provide in-depth information about their perceived functions of playfulness in EFL learning. More specifically, we asked them “what functions of playfulness can you think of while learning English as a foreign language?” The unstructured interview format including its open-ended question could encourage the participants to provide deeper insights into the related points in an exploratory manner (Dörnyei, 2007). It should be mentioned that in case of having no experiences of playfulness, they were free to indicate “no function” for playfulness in the interviews. The interview aimed at giving the participants an overall conception of the target construct and providing them with freedom to think of as many functions of playfulness as possible without limiting their mind to any predefined function or category. Due to the pandemic conditions of the COVID-19 at the time of data collection, all the interviews were conducted online and in the participants’ first language (Persian) to make sure the L2 proficiency did not limit the depth of the information they provided. The interviews were recorded for the data analysis.

### Data Collection

At first, the participants were introduced to the overall meaning of the term, playfulness. This was beyond a lecture and the participants could ask any question for further clarification in order to make sure they were adequately familiarized with the concept in general. Then, the purpose of the present research was revealed to them (i.e., exploring the perceived functions of adult playfulness in L2 learning). Finally, the participants were asked to list as many functions of playfulness as they could think of from their language learning experience. There was no limit set for the number of functions they would name. The participants were free to think of any function that playfulness could play in an actual language learning experience in class. The phrases they provided as the answer are called entries henceforth.

### Data Analysis

In the qualitative content analysis, the conventional content analysis approach was used since the coding categories were directly derived from the interview data. First, interviews were transcribed and read carefully. Then, in order to find tentative codes, three of the authors in this study independently read and analyzed the content of the transcribed interviews. They also searched and highlighted the words which could be considered as key concepts for playfulness. In other words, they identified the emerging and repeated patterns in order to obtain codes through the specification and highlighting the key words reflecting the functions of playfulness. After that, the authors noted, shared and discussed their first impressions of the key concepts of playfulness that they found in the interviews’ data. They continued this process until they specified labels for each code. Later, a frequency count of the codes was conducted and the emerging categories were formed independently. Then, the associations between the codes were identified to come up with unique categories regarding the functions of playfulness. All categorizations were also discussed to solve any point of disagreement (if any). From the initial 38 participants, at last, only the data from 24 could be analyzed and scored as the others were incomplete and could not be used for the final analysis. For example, after reading all the transcripts of interviews, 17 participants perceived language learning very entertaining when they were involved in interactive tasks, solving problems in groups, and using and sharing their knowledge with one another. After finding similar key concepts and assigning codes to each concept, the impressions were shared, the similarities were identified, and differences of the extracted codes were discussed, and similar codes were grouped under one category called cultivating relationships. This process was repeated for other codes and categories. The inter-coder agreement was .91 (Cohen’s K), which is above the suggested threshold level proposed by Miles and Huberman (1994).

## Results

The final analysis of the collected data led to four categories, namely:

• *fun and laughter* (entries related to having fun in learning while using playfulness)
• *creativity* (entries associated with using and creating creative ways to learn English)
• *mastery orientation* (entries related to motivating oneself to master issues and to feel competent)
• *cultivating relationships* (entries related to using inter-personal relationships in improving their English skill)

As seen in Table 1, we found examples for the functions of playfulness in all areas for each category. The total number of entries was 225. Most of the perceived functions of playfulness were ascribed to *the creativity category* (62.22%), followed by *mastery orientation* (17.33%), *cultivating relationships* (15.55%), and *fun and laughter* (4.88%).

**TABLE 1:** Distribution of perceived functions of playfulness in an EFL classroom.

Functions categories Area: EFL learning class	Codes *(frequency* *of each code)*	Total frequency	N
Fun and laughter	*To have fun (10)* *To be cheerful and fool around* *with others (1)*	*11*	*11*
Creativity	*To spend my time in a more interesting way (19)* *To create new things (7)* *To experience new things (19)* *To be creative (21)* *To prevent routine (17)* *To find solutions (14)* *To experience variety (20)* *To keep the excitement (22)* *To explore and learn about target culture (1*)	*140*	*24*
Mastery orientation	*To encourage others (9)* *To motivate myself (13)* *To overcome challenges (13)* *To feel competent (3)* *to enhance memory span (1)*	*39*	*19*
Cultivating relationships	*To interact with others (12)* *To build socialization (12)* *To achieve something together (4)* *To benefit from others’ information (7)*	*35*	*17*

Each of the extracted functions (represented as a category) is elaborated on below with excerpts from the participants’ accounts.

### Fun and Laughter

It has been proposed that the integration of playfulness into the learning environment can lead to deeper learning. Eleven participants shared this belief that fooling around and having fun created a more positive learning environment and could facilitate learning. One participant commented:

I learn better and feel pleased when we watch an interesting movie or listen to a song together in class. In this way I can learn and have fun at the same time. I see that friends can learn better too and enjoy themselves at the same time as well. This is hardly useful at home. But in class having fun together help us learn better. We even sometimes act the funny characters in the movie while watching them and put their names on each other, and teasing each other by such names, haha. (Participant #4, male, 19 years old)

This quotation vividly shows how integrating fun into learning in class can increase language learners’ motivation and can create a kind of learning not expected to be as effective at home. The majority of the participants mentioned that being playful in class brought more excitement to the learning environment and, therefore, increased their enjoyment in class. Another participant stated:

When we are allowed enough time in class to do a puzzle work, telling jokes, or playing a word game together especially when we are too tired to listen to the teacher instructing a new grammar or something like that, we feel so much fun that without noticing the passage of time. (Participant #12, female, 21 years old)

The above-mentioned account points to the gist of playfulness (i.e., the reframing of almost any environment—including those more tiresome in class). In other words, the students were provided with tasks that could effectively help them escape the boring class time and get engaged in interactive activities that were both instructional and motivating. This can be seen as an efficient class management strategy too (on the teacher’s part). When students are bored, they can easily become distracted and refrain from listening to the teacher. Thus, one recommendation could be that teachers replace lecture-based instructions with more interactive tasks (especially multimedia tasks) to reframe the tiresome learning environment and change it to an enjoyable and effective learning context.

Among instances of interactive tasks that the present participants reported in their accounts were word games, puzzles, songs, videos, and other game-like tasks and activities. One participant (#28, female, 20 years old) stated: “Singing along with your favorite singer is really enjoyable and in fact can help you improve your pronunciation.” Thus, EFL learners seem to seize the chance to learn the language in an enjoyable setting through cautiously assigned interactive tasks and activities.

### Creativity

Twenty-four participants found playfulness as a means to create or experience new ways to distance themselves from conventional learning methods and make the learning more exciting and diverse. The most creative ways tried by the participants were watching movies, listening to music, surfing the internet, using social media, games and so forth for specific purposes such as improving their listening comprehension, vocabulary, and accent. Most participants shared this belief that using diverse learning methods was the best way to create variety and escape routines in the learning process. The main reason is that they can improve their English proficiency. In particular, participants described learning different language skills (e.g., speaking, listening, pronunciation, grammar and vocabulary) through challenging tasks in class or as home assignments. See an example of creative class activities to escape boredom and show playful behavior:

This semester, during the last 30 minutes of class time when we are almost all tired and wish to have our books closed, we listen to a song, the incomplete tape-script of which is given to us (by the teacher). It is so much fun that we all forget the time pass. (Participant #10, female, 23 years old)

The above-mentioned account shows how the most expected boring time of class (the last few minutes) can be turned into the most interesting part. It seems that this reframing of the learning environment had a motivating role for students too. Below is another instance of a creative home assignment that managed to highly engage EFL learners and add a spice to the boring activities in their workbook:

The other day, the teacher replaced the vocabulary activities of the work book to a story-writing task. She said we could make a story at home to include all the new words we learned that day and read it in class the following session. It was so fun. We never experienced it before. She said if we create a story out of the words we learn, we never forget them. (Participant #14, female, 25 years old)

This account shows how playfulness can be extended to beyond-class learning and change language learners’ mood during their extended learning at home (or elsewhere). Among the other instances of creativity applied for playfulness purposes in an EFL learning environment are listening to music, watching movies, pop-quizzes, creative games, role-plays, surfing social networks (e.g., YouTube, Instagram, Facebook). The additional benefit of these creative tasks and activities is that they not only manage to change the monotonous mood of the class to an enjoyable one, but can also be motivating for learners, a kind of internal motivation that can come with a sense of internal self-satisfaction. With this respect, a student (Participant #26, male, 21 years old) commented: “I never felt so good at listening before the song-tasks we are doing this semester.” It is reasonable to assume that the creative task of song-writing in class managed to increase the student’s perceived self-efficacy in listening skill.

Multi-media creative tasks and activities for specific purposes (e.g., watching movies or surfing YouTube) have the further benefit of raising the cultural awareness of the target language and the retention of specific expressions and idioms. This is expressed in one participant’s comments: “Watching movies have many advantages. You can enjoy your time, learn new vocabulary and learn about their lifestyle and cultural habits as well.” Another participant (Participant #9, male, 22 years old) mentioned: “Searching YouTube channels and following English-speaking YouTubers help you learn interesting things about their culture and lifestyles.” Movies can provide a source of authentic and varied language. Nearly all participants reported that movies familiarized them with instances of English in authentic situations out of the classroom, especially how to interact in English. This belief was reflected in one of the participants’ remarks:

Movies and TV shows are primary sources of vocabulary acquisition, Watching movies will help you get familiar with popular phrases and words and gives you this chance to learn to hear a native speaker talk at the natural speed. This will help you improve both your listening and speaking skills. You will get the chance to hear how natives pronounce certain words, and to learn every-day conversations. (Participant #17, female, 22 years old)

Thus, it seems that certain tasks and activities could be creatively constructed and applied in (or possibly outside) an EFL class aiming basically to change the boring environment into an enjoyable one. Yet, they can have several considerable benefits too, such as providing access to authentic materials, raising cultural awareness, acting as a source of internal motivation, increasing perceived self-efficacy and creating great chances for thinking beyond the predefined or presumed syllabus or lesson plans and allowing for engagement in more challenging and simultaneously fruitful learning experiences.

### Mastery Orientation

As for the mastery orientation category, 19 participants found functions of playfulness associated with this category and tried to encourage themselves and others to improve their English proficiency through engagement in as many playful activities as possible. One of the best ways to overcome learning obstacles is to challenge oneself to practice English in different situations beyond the routine tasks and activities. Several participants reported that their practicing speaking in online social platforms gave them confidence to speak in face-to-face situations. See an example below:

My online experience of language learning was very challenging at first, but now I feel it really pays off. Most often the cameras are off, which makes the class boring but there are certain facilities like break-out rooms to change the whole atmosphere of the class and enjoy our learning experience. For sure, the brand new online experience of language learning is quite a challenge, the challenge makes it interesting too. It is like a new route toward the same target. (Participant #13, male, 23 years old)

The playful mood learners experienced in certain tasks and activities served a function beyond the mere joy of learning. It seems to have helped them master a good command of certain language skills (e.g., speaking) or area (e.g., vocabulary). See the comment below:

I always kept forgetting the meaning of new words. The old ways I always tried did not help until I came to know a variety of techniques (thanks to the teacher who taught us all) which were both fun and effective. Word puzzles, story construction and online vocabulary builder revolutionized my knowledge of vocabulary. (Participant #30, female, 21 years old)

As inferred from the above-mentioned account, new challenging vocabulary building techniques helped the EFL learner master English vocabulary in an ideal way (at least as perceived by herself). The efficient use of technology to master the required skills of English language seems to be significantly involved in adding variety to the language learning environment and turning the old ways of language learning into a new enjoyable experience. Computer-based and mobile-based applications and websites have opened new horizons to EFL learners, which they come to know about through peers or teachers. Below is a prototypical example:

We are using Encarta as the teacher suggested on both our mobile phones and PCs. It is so much fun to learn almost anything on this encyclopedia. It has also a well-equipped dictionary that gives you synonyms, antonyms, collocations, thesaurus and examples. The kid suite is perfect too. I am gaining a good command of vocabulary. My reading skill has improved too. I enjoy surfing the online version when I am out of class, as a self-study. Thanks to technology, my whole language learning world has changed. (Participant #29, male, 18 years old)

The above-mentioned account shows how the new offline and online sources can be used effectively in revolutionizing old ways along the language learning journey. EFL learners both enjoy the new ways and learn the fact that there is no best way to learn each language skill or area. They are encouraged to go beyond the prescriptive ways of teaching and learning the language.

Another advantage of creating a playful learning environment is to expand the memory span. It can be realized by engagement in activities such as extensive readings, retelling stories, using imagination, and using the surrounding environment to remember as much as you can. It is reflected in one of the participants’ comments: “If I want to practice vocabulary of a specific topic, I use my surrounding environment to enhance my memory. For example, I sit in my car and try to remember as many words as I can and in this way, I learn car-related vocabulary.” Another participant (#35, female, 19 years old) reported: “I use my imagination and relate a word or sentence to a picture to prevent forgetting them.” Also, another student (Participant #7, male, 22 years old) shared a similar belief: “When I hear new words, I try to imagine them in different ways, especially in funny ways so that this method will help me remember that new word easier.”

The above-mentioned comments point to the direct and indirect contribution of a playful learning environment to EFL learners’ mastery of the language skills and areas. As all the instances provided by the interviewees showed, students enjoyed getting engaged in new and challenging tasks and activities to explore new ways of language learning.

### Cultivating Relationships

This category, with interpersonal relationships at heart, can be approached from at least two perspectives: interpersonal relationships among teacher and students or among students together on the one hand, and interpersonal relationships between students and native speakers on the other. The former primarily (but not exclusively) occurs within classroom boundaries and the latter occurs mostly through online sources especially social networks and language learning forums. Instances of both are provided below.

Within the classroom boundary, the participants experienced certain interactive tasks and activities to change students’ mood and revive the whole class. Well-known examples of these tasks and activities in an EFL classroom are free discussions, panel discussions, cooperative problem-solving tasks, Q&As and so on. These interactive and cooperative tasks seem to serve a dual function, engaging all students (even the most conservative) and living up their spirits on the one hand and creating chances for learning the language cooperatively on the other. This dual service can be inferred from the following account:

When we are tired, often at the end of the class, we start nagging and ask the teacher to stop teaching the book and instead have a free discussion. Everybody likes it, not only to escape reading the textbook but also to talk with each other and share ideas. We enjoy it, and we often do it either as a whole-class activity or in groups of 3–4 students. (Participant #12, male, 23 years old)

Another similar account reads:

We always appreciate having more speaking tasks in class. They seem to never tire us. We do not feel how the time passes as we are enthusiastically involved in speaking, and this is always done together with other students. There is a chance to learn from each other and even know each other better. (Participant #31, female, 24 years old)

Thus, cooperative tasks especially speaking tasks in different forms (as mentioned in the interviews) in an EFL classroom can both interest learners and give them a chance to get acquainted with each other and even make friends. The simplest form of conversation often found in all EFL classes (of almost all levels of proficiency) is “greeting.” One or two sample greeting conversations are often provided in the textbook which the students read and are then expected to reframe with their own names to practice in pairs. This is how they learn how to break the ice and open a conversation with others. This is how they learn to establish relationships with others. Certainly, as inferred from the content of interviews, this task is truly enjoyable to EFL learners and they perceive it as the true meaning of communication, and they perceive communication as the primary goal of language learning. With this respect, a participant (#10, female, 20 years old) commented: “We enjoy speaking tasks so much in class because we finally get a chance to use what we’ve learned to communicate with each other. What else should we want from the language?”

Outside the classroom boundary, EFL learners often enjoy chatting with native speakers (and even non-natives) but value the former more. This can be realized in online platforms, chatrooms, social media especially Facebook, forums, and other potential environments to share ideas with native English language speakers. EFL learners often find these communications enjoyable as they believe they can learn a lot from native speakers and test their own communicative competence too. In other words, they seize the chance to realize whether they can communicate effectively with natives or not. If they feel they fail to do so, they may perceive no value in attending the language classes. See a relevant account:

I have a pen-pal to whom I write emails. I am not yet confident enough to chat with him online. But I really enjoy reading his replies. When there is a point I miss, I ask him to clarify it in the next email. But he insists we chat together. I will consider it in near future maybe. But that’s so much fun. I wholeheartedly feel I am using the language I learned and it really works. (Participant #32, male, 20 years old)

From the above-mentioned account, we can conceive that language learners best enjoy themselves when they are communicating directly with a native speaker. They might find it very interesting, motivating, and effective while making friends with a native speaker beyond the classroom setting.

## Discussion

Guided by the advent of positive psychology in SLA domain (MacIntyre and Gregersen, 2012), the present research was an attempt to unravel the perceived functions of adult playfulness in EFL learning domain. Earlier research has highlighted that playfulness is an indicator of positive psychological functioning and this is the first study to investigate what functions the language learners themselves see in playfulness. Based on the interviews, we identified four categories of perceived functions: *Fun and laughter*; *creativity*; *mastery orientation*; and *cultivating relationships*. This converges relatively well with Proyer’s (2014) categories; namely, well-being, humor and laughter, mastery orientation, creativity, relationship, coping strategies, and coping with situations.

A comparison of the categories extracted from the present analysis and the study by Proyer (2014) revealed several links among the categories and the major categories of functions served by adult playfulness in psychology. Interestingly, a major difference with the 2014 study was that considerably fewer language learners chose the “fun and laughing”-category than the lay people from the general public tested there. Again, this underlines to importance of context and environment and the relevance of studying playfulness in specific groups. This also points to the need for a broadening of the playfulness concept: namely, not only seeing humor and entertainment as important correlates, but also components that potentially facilitate learning and stimulate interest (cf. Proyer, 2014, 2017). The *fun and laughter* and *cultivating relationships* categories in the EFL setting can be subsumed under the other-directed facet of playfulness (Proyer, 2017). EFL learner participants provided examples of reframing the negative or boring atmosphere of class by engagement in cooperative tasks and interacting with others. Even outside class, they enjoyed learning English through contacting native English speakers in different forms (e.g., social networking, email correspondence, forums, etc.). Thus, either as self-initiated attempts or guided by their teacher, they added variety to their language experience by involving others into a playful experience.

As in Proyer (2014), being creative seems to be an important component to playfulness (see Proyer et al., 2019 for an overview). Using creative approaches to learning and working with teaching materials may be a positive contributor to successful language acquisition. Proyer (2011) found that greater playfulness relates to better grades among students in a written exam and speculated that this could be related to how the more playful students prepare for the exam (e.g., making the learning experience more interesting; enjoying the challenge, etc.) and/or how they can cope with the stressors during the exam (e.g., being able to distract themselves or finding ways of relaxing during the exam). An interesting follow-up study would be to see how language learners can capitalize on playfulness to make their learning experience more interesting, engaging, and, potentially, also more successful.

Creativity and master orientations as perceived functions of playfulness can be linked to the intellectual function of adult playfulness in the structural model of the construct proposed and confirmed by Proyer (2017). As the participants’ accounts and instances showed, they tried creative tasks and activities (even not integrated in the course syllabus or lesson plans) such as role-plays, puzzle games, pop quizzes and the like to extend their linguistic competence and achieve a good command of different language skills and areas. At the core of the intellectual function of playfulness is deliberate tackling of new ideas and getting involved in complex and challenging tasks and activities (Proyer, 2017). That is, why the two extracted categories of functions in the EFL setting can be integrated into the intellectual function of playfulness in the L2 learning domain.

The results of this study extend Proyer’s (2014) findings that people can experience various functions of playfulness in a multitude of areas in life including language learning context. The functions attributed to playfulness by L2 learners in the current study were also reported in the earlier literature (Proyer, 2014). However, it should be noted that the perceived functions of playfulness in the previous studies were much broader maybe because these studies examined the perceived functions of playfulness among adult laypeople in everyday situations but in the present study, we examined functions of playfulness in a specific context of language learning, leading to a narrower range of functions.

The perceived functions of playfulness identified in this study also lend support to Proyer’s (2017) definition of playfulness as “an individual differences variable that allows people to frame or reframe everyday situations in a way such that they experience them as entertaining, and/or intellectually stimulating, and/or personally interesting” (p. 93). Furthermore, Proyer (2017) sees establishing and maintaining good relationship with others as one essential component of playfulness. That is, the perceived functions of playfulness among L2 learners in our study can be identified within Proyer’s (2017) definition of this concept. That is, our findings confirm Proyer’s (2014) view that playfulness not only facilitates joy and pleasure, but also it has some cognitive components (e.g., *mastery orientation*) that enable people to motivate themselves and others.

The present findings also showed that different functions of playfulness, as perceived by the participants, can create an enjoyable learning environment. This is in tandem with the findings of other studies in the field of education including Zeng et al. (2020). They found that incorporating play in learning, particularly in the form of educational games, can highly increase enjoyment among learners. If the affective filter is lowered, learners can learn through enjoyment. Besides, playfulness is associated with higher self-estimates of ingenuity. This is in agreement with Proyer et al. (2019) who considered creativity as strongly related to playfulness. Lieberman (1977) argued that manifest joy and spontaneity inherent to play can also contribute to other tasks such as those in need of divergent thinking. This resembles what was proposed by the broaden-and-build-theory of positive emotions (e.g., Fredrickson, 2001). The idea was that the experience of positive emotions broadens individuals’ present action and thought repertoire and facilitates building resources. Play and playfulness can facilitate the experience of such emotions (e.g., Panksepp, 1993; Fredrickson, 2001) and, thereby, can also facilitate creativity.

In the present study, EFL learners used different learning tasks and activities such as watching movies, listening to songs, surfing the net, using social media, playing games, and so forth, to incorporate fun into learning, to escape learning routines, and to integrate more creative ways into their language learning experience. In the L2 learning context, different tools were employed to add variety and innovation and to increase creativity and accordingly promote language learning. Broadly speaking, this is consistent with Proyer (2011) who concluded that playfulness can be associated with academic success. More specifically, this is consistent with Maslavchuk (2019) who perceived the incorporation of enjoyable tasks and activities such as watching movies as a source of both entertainment and education in different settings. As also pinpointed by Ismaili (2013), creative multimedia tasks such as watching movie can enhance English language proficiency due to the provision of authentic input and simulation of a natural context for language learning. The authenticity privilege was stated in the present findings too, as the interviewees enjoyed the authenticity inherent to the movies they watched (discussed in the creativity category in the results section).

The authentic source of learning can lead to better vocabulary acquisition (Webb, 2010; Ashcroft et al., 2018; Feng and Webb, 2020) and can also increase cultural awareness of the target language. This point was also found in the present EFL learner participants’ accounts of the creativity category. This finding is in line with Zhang (2013) who indicated that watching movies highly increased students’ awareness of the target culture. As the present findings showed, movies and music were potent sources of triggering interest in language learning. This is in line with Luo (2004) and Zulfahmi and Nikmah (2020) who concluded that movies can trigger interest in English language learning. Movies and music (providing audio- and visual authentic input) were also considered as a source of enhancing other language skills such as listening, speaking, grammar and pronunciation (reported in the mastery orientation category of playfulness in the results section). This is in line with different studies that also confirmed the effective role of movies and music in enhancing listening skill (e.g., Qiu and Huang, 2012; Kumar and Sandaran, 2018) and speaking acquisition (e.g., Porcel, 2010), grammar (e.g., Mushtaq and Zehra, 2016) and pronunciation mastery.

The findings of this study, as perceived by the participants, indicated that to improve their mastery in English language skills, technology (especially computers and mobile phones) can be regarded as potential sources of showing playful manners to change the mood of the whole class and to personalize learning. This is consistent with the findings of Ahmed (2020), who also concluded that mobile technology is an effective way to improve learning. Computer assisted language learning (CALL) and mobile assisted language learning (MALL) domains which have hosted quite many investigations in recent years can, from now on, attend more specifically to the playfulness construct in language learning too, as new technologies can be effectively used to change negative or tedious learning environment to a positive and enjoyable one, as ratified by the present findings. Similarly, games and especially guessing games were found in the present study to be as a playful source of learning that highly improved language learners’ speaking skill and vocabulary acquisition. This is in line with who concluded that guessing-game techniques highly improved L2 learners’ speaking skill.

The overall findings pointed to the fact that language learners’ emotions are significantly involved in the quality and quantity of language learning, as also acknowledged by many researchers in applied linguistics. At the heart of playfulness is the ability to (re-)frame almost any situation (including those high in boredom) in a way to make the situation more entertaining, interesting, and/or stimulating (e.g., by eliciting positive emotions such as enjoyment and motivation). However, it has been scarcely investigated in the language learning domain compared to the recent attention to positive emotions in the field of applied linguistics (see Dewaele et al., 2019). The present research was a preliminary study of the concept of adult playfulness in EFL learning context. This concept still needs further investigation (with larger samples) to become more refined and representative of the target research population. The present findings provided insightful information about how language learners experienced the reframing of conventional, routine and probably tedious language learning into an enjoyable, creative and productive one. The categories of the functions attributed to playfulness and the similarities found with those in general psychology resulted in a binary re-categorization of functions, other-directed and intellectual, in EFL learning domain. This two-dimensional conceptual definition of the construct in EFL setting requires empirical data for construct validation though.

## Conclusion

Given the growth of positive psychology in SLA and the focus on both negative and positive emotions in this line of research, we maintain that the concept of playfulness can contribute to the expansion of this research domain. With this in mind, we aimed to explore the functions of playfulness among adult learners of English as a foreign language. The findings indicated that adult EFL learners’ perceived that playfulness can be divided into four categories; namely, humor and laughter, cultivating relationships, creativity and mastery orientation. In association with the related literature on adult playfulness in psychology, the first categories can be further subsumed as the other-directed function of playfulness, while the next two can be integrated into one category of the intellectual function. From a pedagogical perspective, the findings of this study can provide EFL teachers with insights into how to help their learners frame learning situations and classroom tasks into enjoyable and entertaining ones.

The findings of the current study shed light on how adult language learners see playfulness. Based on these findings, future research can formulate and examine hypotheses concerning the nomological net of playfulness in language learning context. As suggested by prior research, playfulness might be positively related to joy (Proyer and Brauer, 2018), intrinsic motivation (Proyer, 2012), and negatively related to anxiety and boredom (Barnett, 2012). Future research can test all these associations empirically in the context of language learning. The findings of this study can have practical implications in the area of positive interventions that aim at developing positive emotions, behaviors, and cognitions (Proyer, 2014). These interventions can make a unique contribution to an individual’s wellbeing. The difference between play and work (e.g., drudgery) as mentioned before is a matter of attitude, rather than what people actually do. For example, taking a walk or solving a math problem might be a very enjoyable play for one person while a very hard and boring work for another person (Bateson et al., 2013). Doing a serious work like learning a second language playfully can lead to healthy and positive consequences such as innovation, creativity, and less stress and anxiety, all of which are necessary for learning a new language.

However, it must be mentioned that participants in this study used self-reports to list the perceived functions of playfulness, and that this type of data elicitation may be subject to social desirability and hence, may not display what people actually do when they use playfulness. Further, the present research was preliminary in exploring the conceptual definition of the construct of playfulness in the EFL learning domain. Operationalization of the construct requires further empirical data exploration, expected in near future to contribute to the positive psychology domain in SLA.

## Data Availability Statement

The raw data supporting the conclusions of this article will be made available by the authors, without undue reservation.

## Ethics Statement

Ethical review and approval was not required for the study on human participants in accordance with the local legislation and institutional requirements. Written informed consent for participation was not required for this study in accordance with the national legislation and the institutional requirements.

## Author Contributions

All authors listed have made a substantial, direct, and intellectual contribution to the work, and approved it for publication.

## Conflict of Interest

The authors declare that the research was conducted in the absence of any commercial or financial relationships that could be construed as a potential conflict of interest.

## Publisher’s Note

All claims expressed in this article are solely those of the authors and do not necessarily represent those of their affiliated organizations, or those of the publisher, the editors and the reviewers. Any product that may be evaluated in this article, or claim that may be made by its manufacturer, is not guaranteed or endorsed by the publisher.
